# Prevalence and Trends of Post–COVID-19 Sequelae Among U.S. Adults With Prior COVID-19 Infection, 2022–2024

**DOI:** 10.7759/cureus.116792

**Published:** 2026-09-23

**Authors:** Ashish Bhargava, Susan Szpunar

**Affiliations:** 1 Infectious Disease, Henry Ford St. John Hospital, Detroit, USA; 2 Biostatistics, Henry Ford St. John Hospital, Detroit, USA

**Keywords:** long covid, national health interview survey, pasc, postinfection risk, prevalence

## Abstract

Background: Post-acute sequelae of SARS-CoV-2 infection (PASC) affect a substantial proportion of U.S. adults, involving multiple organ systems and resulting in significant disability and diminished quality of life. Population-level PASC estimates include individuals without prior COVID-19 infection and therefore do not accurately reflect postinfection risk required for effective risk stratification. We estimated the prevalence and trends of PASC among U.S. adults with prior COVID-19.

Methods: We analyzed data from the National Health Interview Survey (2022-2024), a nationally representative survey of the U.S. civilian noninstitutionalized population. Adults reporting prior COVID-19 infection were asked whether they ever experienced symptoms lasting ≥3 months that were not present before infection; those answering yes were classified as having PASC. Weighted analyses accounted for the complex survey design.

Results: Among 42,872 adults with prior COVID-19, 6,822 reported symptoms lasting three months or longer, indicating PASC. Prevalence declined from 19.7% in 2022 to 15.1% in 2023 and 13.7% in 2024 (P<0.001 for 2022 vs subsequent years). Despite this decline, more than 21 million U.S. adults reported having experienced PASC in 2023 and 2024. Prevalence was higher among women and adults aged 35-64 years than among men and younger or elderly individuals. A lower prevalence was observed among Asian adults, whereas a higher prevalence was noted among American Indian, Alaska Native, and multiracial adults.

Conclusions: Despite declining prevalence, PASC remains common among U.S. adults with prior COVID-19. These nationally representative postinfection estimates provide clinically relevant risk information to guide patient counseling, health system planning, and ongoing surveillance.

## Introduction

Post-acute sequelae of SARS-CoV-2 infection (PASC) involves over 200 symptoms affecting approximately 10 organ systems, most commonly fatigue, post-exertional malaise, cognitive impairment, and dyspnea [1]. It is associated with significant disability, diminished quality of life, and increased healthcare utilization [1-4]. The clinical course is variable and frequently relapsing-remitting; full recovery is uncommon beyond six months, and many individuals experience persistent symptoms for years [1]. Currently, no validated biomarkers or formal diagnostic criteria exist for PASC, and no universally accepted case definition has been established. The World Health Organization (WHO) Delphi consensus describes PASC as new or persistent symptoms developing within three months of infection, persisting for at least two months, and not explained by an alternative diagnosis. The Centers for Disease Control and Prevention (CDC) characterizes long COVID as a chronic condition that continues or emerges at least three months after SARS-CoV-2 infection, encompassing a wide range of new, returning, or ongoing health problems [5,6]. The National Health Interview Survey (NHIS) uses the criterion of symptoms persisting for three months or longer after infection as the case definition in this study. Population-level estimates from the NHIS and the Medical Expenditure Panel Survey (MEPS), which are population-based samples, indicate that between six and nine percent of U.S. adults have experienced PASC [7,8]. However, these estimates include individuals without prior COVID-19 infection and therefore do not accurately reflect the post-infection risk required for effective risk stratification. Nationally representative, multi-year estimates of post-infection PASC remain unavailable, and consistent demographic disparities have not been identified across survey years.

Our study makes four significant contributions. First, it presents the first three-year, nationally representative estimate of PASC prevalence among adults with prior COVID-19 infection, yielding post-infection-specific rates. Second, by utilizing three consecutive annual surveys, the study examines whether PASC prevalence has stabilized or continued to decline in the post-Omicron era. Third, this analysis evaluates the consistency of demographic disparities by sex, age, and race across all three survey years, thereby strengthening the evidence base beyond the limitations of a single cross-sectional study. Fourth, the NHIS is the only publicly available, annually repeated, nationally representative United States survey that has included PASC-specific questions since 2022. As a result, these analyses provide a primary source of nationally generalizable, trend-level PASC surveillance data.

The objective of this study was to estimate the annual prevalence of ever-reported PASC among U.S. adults with prior COVID-19 infection using nationally representative NHIS data from 2022 to 2024, and to examine trends and demographic differences in prevalence across these survey years.

## Materials and methods

This study was conducted in accordance with the Declaration of Helsinki. It uses publicly available, completely de-identified data and is IRB-exempt; patient consent was not required. We analyzed data from the NHIS, years 2022-2024, a nationally representative cross-sectional survey of the U.S. civilian non-institutionalized population [9,10]. This survey is conducted annually by the National Center for Health Statistics (NCHS) using a multistage stratified sampling method. The NHIS methodology is detailed by the NCHS [10]. Beginning in 2022, NCHS prospectively incorporated COVID-19-specific questions into the NHIS Sample Adult questionnaire to capture PASC at the population level; this was not a retrospective repurposing of existing data. Trained NCHS field interviewers administer the survey in person using computer-assisted personal interviewing (CAPI). The NCHS website provides public access to all data and the complex sampling plan file, which enables full replication [9,10].

Adults ages 18 years and older who completed the Sample Adult questionnaire from 2022 to 2024 and answered the COVID-19 items were eligible. Respondents reporting a history of COVID-19 comprised the denominator for estimating prevalence among adults with prior infection. Prior COVID-19 infection was defined using the NHIS variable CVDDIAG_A in 2022; EVERCOVD_A in 2023-2024, which asks, "Have you ever had COVID-19?" Respondents were instructed to include diagnoses by a doctor or other healthcare professional, positive antibody or swab testing, and symptomatic illness following close contact with a confirmed case. PASC was defined using LONGCVD_A in 2022-2023 and LONGCOVD2_A in 2024, "which asks whether the respondent ever experienced symptoms lasting three months or longer not present before COVID-19?". The questionnaire identified long-term symptoms as including, but not limited to, fatigue, cognitive impairment ("brain fog"), dyspnea, joint or muscle pain, heart palpitations, chest pain, and changes in taste or smell. Respondents who answered yes were classified as having PASC. Because this variable captures a cumulative ever-measure, respondents who have since recovered from prolonged symptoms may still be included. This approach differs from the NHIS variable for currently active PASC (SYMPNOW_A in 2022-2023; SYMPNOW1_A in 2024), which was not used in this analysis. Respondents with missing, refused, or unknown responses to either variable were excluded. We analyzed demographic variables: sex (SEX_A), race (RACEALLP_A), Hispanic ethnicity (HISP_A), marital status (MARITAL_A), age (AGEP_A) and age group (Age_recode). Survey weights (WTFA_A) were applied throughout.

Data from 2022, 2023, and 2024 NHIS surveys were downloaded and analyzed, with weighting applied to account for the complex sampling design. Data were analyzed using descriptive statistics, the chi-squared test and general linear models using the Complex Samples functions in SPSS v. 30.0.1.

## Results

In the NHIS, a sample adult questionnaire conducted from 2022 to 2024, 42,872 participants reported a prior COVID-19 infection. Of these individuals, 6,822 experienced symptoms lasting three months or longer, leading to their classification as having PASC. In 2022, 1,797 adults with prior COVID-19 reported PASC, corresponding to a weighted estimate of approximately 17.6 million U.S. adults and a prevalence of 19.7% among adults with prior COVID-19. In 2023, 2,398 adults reported PASC, corresponding to approximately 21.3 million adults and a prevalence of 15.1% among adults with prior COVID-19. In 2024, 2,627 adults reported PASC, corresponding to approximately 21.3 million adults and a prevalence of 13.7% among adults with prior COVID-19. Prevalence differed significantly between 2022 and the other years, but not between 2023 and 2024 (Figure 1).

**Figure 1:**
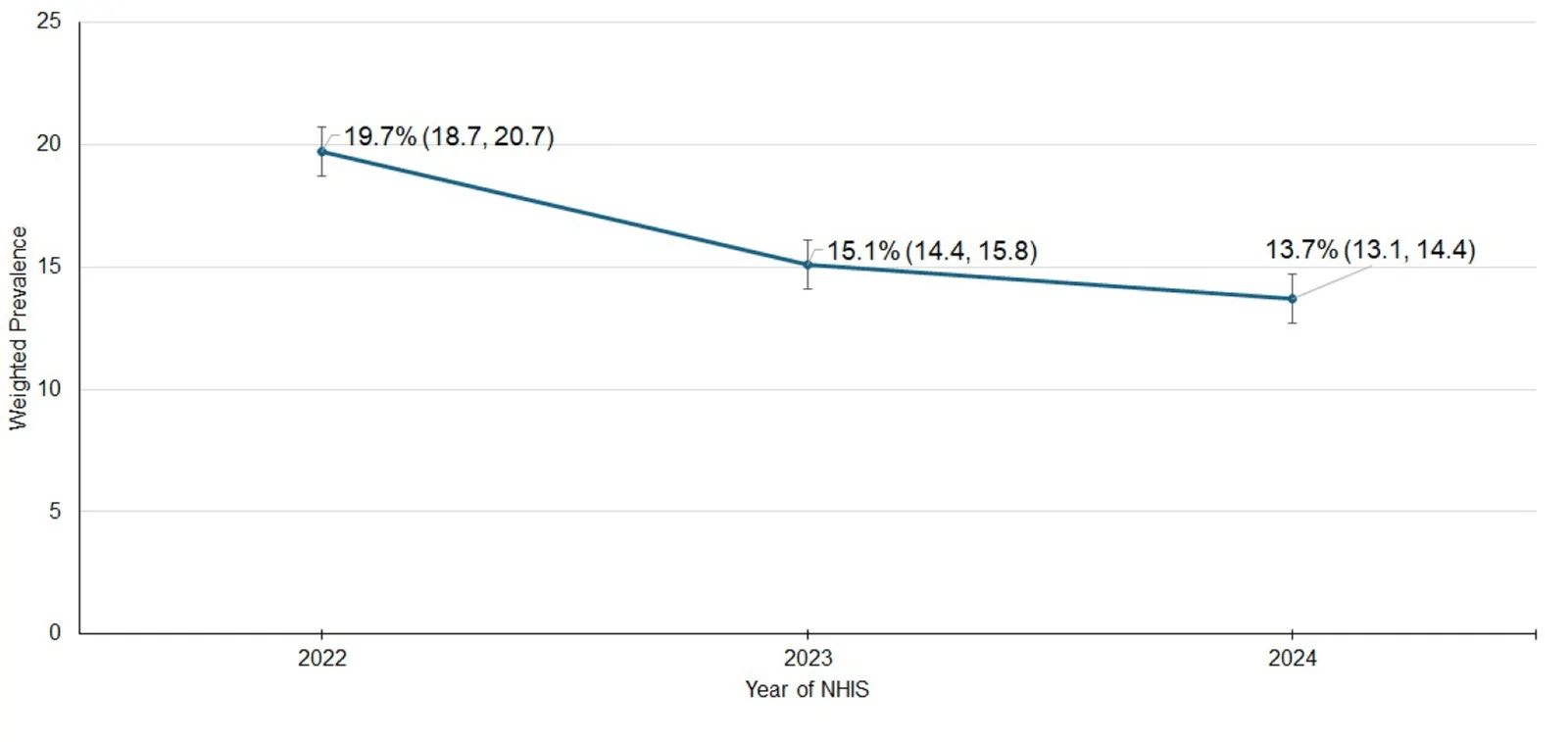
Weighted prevalence (95% confidence interval) of patients reported to have PASC. PASC: Post-acute sequelae of SARS-CoV-2

The mean (SE) age was 48.2 (0.12) years, and it was similar across survey years with no statistically significant differences by year. In 2022, PASC prevalence varied by age group (P < .001), with the highest estimates observed among adults ages 35-49 years (21.8%; 95% CI, 20.0-23.8) and 50-64 years (22.7%; 95% CI, 20.6-24.9), compared with 16.2% (95% CI, 14.6-17.9) among adults ages 18-34 years and 19.1% (95% CI, 17.2-21.3) among those aged ≥65 years (Table 1).

**Table 1:** Prevalence of PASC by demographic characteristics among U.S. adults with prior COVID-19 infection for the year 2022 (NHIS). Abbreviations: PASC: Post-acute sequela of SARS-CoV-2; CI: Confidence interval; AIAN: American Indian or Alaska Native; NIHS: National Health Interview Survey.

Characteristics by Year 2022	Patients with PASC (N =1797) Weighted percentages (95% CI)	Patients without PASC (N=7149) Weighted percentages (95% CI)	F (Degree of Freedom df1, df2) = XX. XX	p-value
Age, Y				
18-34	16.2 (14.6–17.9)	83.8 (82.1–85.4)	F (2.9, 1658.7) = 11.71	<0.001
35-49	21.8 (20.0–23.8)	78.2 (76.2–80.0)		
50-64	22.7 (20.6–24.9)	77.3 (75.1–79.4)		
≥ 65	19.1 (17.2–21.3)	80.9 (78.7–82.8)		
Sex				
Male	15.5 (14.2–16.9)	84.5 (83.1–85.8)	F (1, 571) = 57.67	<0.001
Female	23.3 (21.9–24.8)	76.7 (75.2–78.1)		
Race				
White	19.8 (18.7–20.9)	80.2 (79.1–81.3)	F (4.7, 2706.2) = 5.50	<0.001
Black	19.2 (16.3–22.4)	80.8 (77.6–83.7)		
Asian only	9.1 (6.6–12.3)	90.9 (87.7–93.4)		
AIAN only	26.7 (17.3–38.7)	73.3 (61.3–82.7)		
AIAN and any other group	24.7 (15.7-36.6)	75.3 (63.4-84.3)		
Other single and multiple races	25.3 (17.0-35.9)	74.7 (64.1-83.0)		
Ethnicity				
Hispanic	21.1 (18.8–23.9)	78.8 (76.1–81.2)	F (1, 571) = 1.97	0.20
Non-Hispanic	19.3 (18.3–20.4)	80.7 (79.6–81.7)		
Marital Status				
Married	19.4 (18.1–20.7)	80.6 (79.3–81.9)	F (1.9, 1125.7) = 0.82	0.44
Living with a partner	21.5 (18.2–25.1)	78.5 (74.9–81.8)		
Neither	19.3 (17.8–20.9)	80.7 (79.1–82.2)		

In both 2023 and 2024, similar patterns were observed, with a higher prevalence among adults ages 35 to 49 and 50 to 64 years than among those ages 18 to 34 and those over 65 years. The overall absolute prevalence, however, declined across all age groups during this period (Table 2-3). 

**Table 2:** Prevalence of PASC by demographic characteristics among U.S. adults with prior COVID-19 infection for the year 2023 (NHIS). Abbreviations: PASC: Post-acute sequela of SARS-CoV-2; CI: Confidence interval; AIAN: American Indian or Alaska Native; NIHS: National Health Interview Survey.

Characteristics by Year 2023	Patients with PASC (N=2398) Weighted percentages (95% CI)	Patients without PASC (N=12893) Weighted percentages (95% CI)	F (Degree of Freedom df1, df2) = XX.X	p value
Age, Y				
18-34	13.3 (12.1–14.7)	86.7 (85.3–87.9)	F (2.9, 1749.4) = 6.47	<0.001
35-49	16.0 (14.6–17.4)	84.0 (82.6–85.4)		
50-64	16.9 (15.6–18.3)	83.1 (81.7–84.4)		
≥ 65	14.4 (13.1–15.7)	85.6 (84.3–86.9)		
Sex				
Male	11.9 (11.1–12.9)	88.1 (87.1–88.9)	F (1, 610) = 80.34	<0.001
Female	17.8 (16.9–18.8)	82.2 (81.2–83.1)		
Race				
White	15.2 (14.5–16.1)	84.8 (83.9–85.5)	F (4.8, 2905.4) = 5.23	<0.001
Black	14.5 (12.5–16.8)	85.5 (83.2–87.5)		
Asian only	8.7 (6.8–11.1)	91.3 (88.9–93.2)		
AIAN only	18.5 (13.6–24.7)	81.5 (75.3–86.4)		
AIAN and any other group	17.7 (11.4–26.5)	82.3 (73.5-88.6)		
Other single and multiple races	17.9 (12.4–25.2)	82.1 (74.8-87.6)		
Ethnicity				
Hispanic	16.5 (14.9–18.4)	83.5 (81.6–85.1)	F (1, 610) = 3.69	0.06
Non-Hispanic	14.7 (14.0–15.5)	85.3 (84.5–86.0)		
Marital Status				
Married	14.8 (13.9–15.7)	85.2 (84.3–86.1)	F (1.9, 1198.2) = 0.85	0.43
Living with a partner	15.2 (13.1–17.7)	84.8 (82.3–86.9)		
Neither	15.7 (14.6–16.9)	84.3 (83.1–85.4)		

**Table 3:** Prevalence of PASC by demographic characteristics among U.S. adults with prior COVID-19 infection for the year 2024 (NHIS) Abbreviations: PASC: Post-acute sequela of SARS-CoV-2; CI: Confidence interval; AIAN: American Indian or Alaska Native; NIHS: National Health Interview Survey

Characteristics by Year 2024	Patients with PASC (N =2627) Weighted percentages (95% CI)	Patients without PASC (N=16008) Weighted percentages (95% CI)	F (Degree of Freedom df1, df2)	p-value
Age, Y				
18-34	12.9 (11.7–14.2)	87.1 (85.8–88.3)	F (2.9, 1765.2) = 4.18	0.006
35-49	14.9 (13.7–16.1)	85.1 (83.9–86.3)		
50-64	14.6 (13.4–15.8)	85.4 (84.2–86.6)		
≥ 65	12.4 (11.3–13.6)	87.6 (86.4–88.7)		
Sex				
Male	11.3 (10.5–12.2)	88.7 (87.8–89.5)	F (1, 610) = 54.47	<0.001
Female	15.9 (15.0–16.8)	84.1 (83.2–85.0)		
Race				
White	14.0 (13.3–14.8)	86.0 (85.2–86.7)	F (4.9, 3010.7) = 6.61	<0.001
Black	14.6 (12.6–16.8)	85.4 (83.2–87.4)		
Asian only	8.4 (6.7–10.6)	91.6 (89.4–93.3)		
AIAN only	22.6 (15.8-31.4)	77.4 (68.6-84.2)		
AIAN and any other group	21.5 (15.0-29.8)	78.5 (70.2-85.0)		
Other single and multiple races	14.2 (10.4-16.9)	85.8 (81.1-89.6)		
Ethnicity				
Hispanic	13.9 (12.4–15.5)	86.1 (84.5–87.6)	F (1, 610) = 0.04	0.83
Non-Hispanic	13.7 (13.0–14.5)	86.3 (85.5–87.0)		
Marital Status				
Married	13.2 (12.4–14.1)	86.8 (85.9–87.6)	F (2.0, 1219.1) = 4.46	0.01
Living with a partner	16.6 (14.4–19.0)	83.4 (81.0–85.6)		
Neither	14.0 (12.9–15.2)	86.0 (84.8–87.1)		

Over the years of the survey, the prevalence was significantly higher among women than among men. In 2022, the prevalence was 23.3% (95% CI, 21.9-24.8) among women compared with 15.5% (95% CI, 14.2-16.9) among men (p <0.001). Similar differences were observed in 2023 (17.8% vs 11.9%) and 2024 (15.9% vs 11.3%) (both p <0.001) (Table 1-3).

In 2022, the prevalence of PASC was lower among Asian adults (9.1% [95% CI, 6.6-12.3]) compared to White adults (19.8%; 95% CI, 18.7-20.9) and Black adults (19.2%; 95% CI, 16.3-22.4) (p < 0.001). The prevalence was higher among American Indian or Alaska Native adults (26.7%; 95% CI, 17.3-38.7) and among multiracial adults (24.7%; 95% CI, 15.7-36.6). In 2023 and 2024, similar patterns were observed, with Asian adults consistently showing lower prevalence than other racial groups.

No consistent ethnic differences were observed across the various survey years. In 2022, the prevalence among Hispanic adults was 21.1% (95% CI: 18.8-23.9), while it was 19.3% (95% CI: 18.3-20.4) among non-Hispanic adults (p = 0.20). Similarly, in 2024, the differences were not statistically significant (p=0.83).

In both 2022 and 2023, prevalence rates did not differ by marital status. However, in 2024, adults living with a partner had a higher prevalence (16.6%; 95% CI, 14.4-19.0) than married adults (13.2%; 95% CI, 12.4-14.1) (p=0.01).

## Discussion

In our nationally representative analysis, the prevalence of postinfection PASC among U.S. adults declined from 19.7% in 2022 to 13.7% in 2024. In 2022, almost one in five adults reported symptoms lasting three months or more, but by 2024, this number fell to one in seven. Despite the decline, significant demographic disparities by age, sex, and race persisted across all three survey years.

In our study, the observed decline in prolonged symptoms over time is consistent with emerging evidence indicating a lower risk of PASC in later pandemic phases [11-14]. This trend may reflect increasing population immunity from vaccination and prior infection, the emergence of new viral variants, and potentially less severe illness during subsequent Omicron-dominant waves [8]. However, the cross-sectional design does not allow for confirmation of these mechanisms [8]. More than 21 million adults reported having PASC in both 2023 and 2024, despite an overall decline from 2022. By restricting the denominator to individuals with prior infection, our estimates provide a direct measure of postinfection risk, which is substantially higher than population-wide prevalence estimates of 6%-9% reported in national surveys [7,8]. This distinction matters clinically for risk stratification and patient counseling after SARS-CoV-2 infection.

Our analysis revealed consistent demographic patterns across all surveyed years. Women and adults between 35 and 64 had higher PASC rates than men and younger or elderly individuals. This finding is consistent with earlier national reports and meta-analyses that identify being female and middle-aged as important risk factors for PASC [15-17]. The reasons for these differences are still unclear. They may involve differences in immunological responses, such as stronger IgG antibody production and effects linked to X chromosome genes, along with hormonal factors, healthcare-seeking behaviors, and the burden of comorbidities [17,18]. Higher prevalence rates among American Indian, Alaska Native, and multiracial adults in our study correspond with recent findings showing a 21% prevalence of post-COVID-19 conditions in AI/AN adults, with 37% reporting increased functional limitations post-infection [19]. Our study also noted inconsistent differences in Hispanic ethnicity over the years, consistent with variable associations reported in the literature [20,21]. Differences by marital status were not consistently observed across survey years. In 2024, adults living with a partner showed a higher prevalence than married adults. However, this trend did not appear in previous years, so it should be viewed with caution. Observed demographic differences represent unadjusted associations that could be influenced by socioeconomic status, healthcare access, and comorbidity burden. Further research is necessary to clarify these relationships.

More than 21 million adults reported experiencing PASC in 2023 and 2024, demonstrating that the demand for post-COVID care infrastructure remains significant despite a reduction in overall prevalence. Persistent demographic disparities by sex, age, and race highlight the need for targeted interventions, especially for women of working age and American Indian and Alaska Native populations, who experienced higher burdens in each of the three survey years. Postinfection-specific prevalence estimates can inform clinical counseling more precisely than general population figures. Our study found that approximately 1 in 7 adults with prior COVID-19, nearly 1 in 6 women, and 1 in 5 middle-aged adults reported PASC, substantially higher than population-wide estimates of 6%-9% [7,8]. These results indicate that women, adults aged 35-64 years, and American Indian and Alaska Native individuals constitute subgroups for whom post-infection surveillance may be particularly warranted. However, this study's observational design limits conclusions about the clinical impact of targeted follow-up strategies.

This study's strengths include a large, nationally representative dataset spanning three years, which improves trend evaluation and generalizability, along with consistent measurement for valid comparisons. This study's limitations include reliance on self-reported symptoms without formal medical evaluation, which may introduce recall bias, and a lack of data on institutionalized adults. The prior infection variable is based on self-reported infection through clinician diagnosis, laboratory testing, or symptoms after exposure. This may misclassify some cases because it does not require laboratory confirmation. The survey cannot distinguish PASC from other post-viral syndromes because it does not include validated diagnostic tests. Additionally, NHIS does not record the number of infections, limiting assessment of reinfection. Limited information on vaccination status, treatment, and comorbidities further constrains understanding of underlying mechanisms. The cross-sectional design precludes determination of whether observed declines in prevalence reflect recovery or reduced incidence and prevents causal inference. All observed demographic differences reflect unadjusted associations that may be confounded by socioeconomic status, access to healthcare, and comorbidity burden, warranting further research.

## Conclusions

To summarize, the prevalence of PASC among U.S. adults with prior COVID-19 declined substantially from 2022 to 2024, although the burden remains substantial in the Omicron era. A substantial number of U.S. adults continue to be affected, with considerable differences across sex, age, and race. Women, middle-aged individuals, and American Indian/Alaska Native groups have consistently higher PASC prevalence. The postinfection-specific estimates are significantly higher than those observed in the general population and may provide valuable guidance for risk stratification and patient counseling after SARS-CoV-2 infection. Ongoing surveillance is necessary to assess whether the observed plateau constitutes a minimum threshold or signals the onset of a sustained decline, thereby informing clinical recommendations and resource allocation as SARS-CoV-2 evolves.
